# Molecular characterisation of hepatitis B virus in HIV-1 subtype C infected patients in Botswana

**DOI:** 10.1186/s12879-015-1096-4

**Published:** 2015-08-13

**Authors:** Motswedi Anderson, Simani Gaseitsiwe, Sikhulile Moyo, Matthijs J. C. Wessels, Terence Mohammed, Theresa K. Sebunya, Eleanor A. Powell, Joseph Makhema, Jason T. Blackard, Richard Marlink, Max Essex, Rosemary M. Musonda

**Affiliations:** Botswana Harvard AIDS Institute Partnership, Gaborone, Botswana; Department of Biological Sciences, University of Botswana, Gaborone, Botswana; Department of Immunology and Infectious Diseases, Harvard School of Public Health, Boston, USA; University of Cincinnati College of Medicine, Cincinnati, USA

## Abstract

**Background:**

Hepatitis B virus (HBV) is a major global health problem especially in sub-Saharan Africa and in East Asia. Ten hepatitis B virus genotypes have been described that differ by geographic distribution, disease progression, and response to treatment. Escape mutations within the surface open reading frame (ORF) affect HBV antigenicity leading to failures in diagnosis, vaccine and hepatitis B immunoglobulin therapy. However, the molecular characteristics of HBV in Botswana, a highly endemic country, are unknown. We describe the molecular characteristics of HBV and prevalence of escape mutants among HIV/HBV coinfected individuals Botswana.

**Methods:**

DNA was extracted from archived plasma samples from 81 HIV/HBV co-infected participants from various clinical studies at the Botswana Harvard AIDS Institute Partnership. A 415 base pair (bp) fragment of the polymerase gene was amplified by semi-nested PCR. In a subset of samples, a 2100 bp fragment was amplified. The PCR product was genotyped using Big Dye sequencing chemistry and the sequences were analysed for genotypes and mutations.

**Results:**

Of the 81 samples included, 70 (86 %) samples were successfully genotyped. Genotype A was found in 56 (80 %) participants, D in 13 (18.6 %), and 1 (1.4 %) was genotype E. Escape mutations previously linked with failure of diagnosis or escaping active vaccination and passive immunoglobulin therapy were detected in 12 (17.1 %) participants at positions 100, 119, 122, 123, 124, 126, 129, 130, 133, 134 and 140 of the S ORF. Genotypes and escape mutations were not significantly associated with aspartate aminotransferase (AST), alanine aminotransferase (ALT) and AST platelet ratio index (APRI).

**Conclusion:**

Genotypes A, D and E were found in this cohort of HIV coinfected patients in Botswana, consistent with the findings from the sub-Saharan Africa region. Some escape mutations which have previously been associated with diagnosis failure, escaping vaccine and immunoglobulin therapy were also observed and are important in guiding future policies related to vaccine implementation, therapeutic guidelines, and diagnostic guidelines. They are also important for identifying patients who are at an increased risk of disease progression and to choose optimal therapy. Future research should focus on determining the clinical significance of the different HBV genotypes and mutations found in this population.

## Background

The Hepatitis B virus (HBV) still remains a global health problem especially in sub-Saharan Africa and East Asia despite the availability of a safe and effective vaccine [1]. It is estimated that there are 240 million hepatitis B chronic carriers worldwide [1, 2] and it accounts for 780,000 deaths per year due to acute infections, cirrhosis of the liver, and hepatocellular carcinoma (HCC) [1].

Hepatitis B, a prototype of the family *Hepadnaviridae,* consists of a 3.2 kb partially double-stranded DNA arranged in four overlapping open reading frames (ORFs) [3, 4]. The open reading frames are the polymerase [3–5], surface [3, 6, 7], precore/core and the X gene [3, 6, 8, 9]. HBV replicates via reverse transcriptase, an enzyme which has no proof-reading capabilities; hence, nucleotide misincorporations are more common than in other DNA viruses [3]. This has led to the emergence of 10 genotypes A–J [10, 11] classified according to whole genome nucleotide divergence of >7.5 % [12–15]. Genotypes A-D and F have been further classified into subgenotypes [16] based on nucleotide divergence of 4 to 7.5 % [12]. These genotypes and subgenotypes have been shown to have a distinct geographic distribution [12, 17] which might be due to human migration after infection [18]. Genotype A is predominant in Western Europe, Africa and Asia; Genotypes B and C in East Asia; D has a worldwide distribution but is mostly in the mediterranean region; E is prevalent in West and Central Africa; F in South and Central America; G and H in Europe and Japan; I in Vietnam and Laos; J in Japan [12, 19–21]. The predominant genotypes in Africa are A, D and E [22]. The clinical significance of the genotypes has also been demonstrated by several studies [23, 24] which might be due to differences in pathogenesis between genotypes [25]. They have been shown to differ according to the course of disease, development of mutations, and response to antiviral therapy [26–28]. The information on genotypes is important in identifying patients who are at an increased risk of disease progression and in choosing optimal therapy [25].

The hallmark of hepatitis B diagnosis is based on detecting the hepatitis B surface antigen (HBsAg). This region consists of the major hydrophilic region (MHR), an antigenic structure which spans from codon 99 to 169 [29] within which there is the ‘a’ determinant, the most antigenic part of the S gene (amino acid 120 to 147) [30–33]. The ‘a’ determinant is the main target for neutralising antibodies used in active, passive immunisation and in diagnostic assays [34]. Mutations within this region might affect its antigenicity leading to no or weak reactivity with serological assays [29, 35]. They might also lead to the virus escaping the antibodies produced during active vaccination and immunoglobulin therapy [29, 35]. The information on escape mutations is important for guiding policy when developing future vaccines, therapy strategies and diagnostic kits. Also, different diagnostic approaches and treatment strategies might be needed for different regions. HBV often requires lifelong treatment by reverse transcriptase inhibitors, and this might be hampered by development of resistance mutations.

In 2013 the World Health Organization (WHO) issued a statement to its member states, including Botswana, on the global policy report on the prevention and control of viral hepatitis: “Thus, global efforts to make hepatitis a public health priority need to be transformed into prevention and control strategies that are tailored to specific conditions at the national and sub-national levels"  [36]. However,  Botswana, a country with HBV/HIV co-infection prevalence ranging from 4 to 10.6 % [37, 38], has scanty data on hepatitis B prevalence and the molecular characteristics of the hepatitis B virus circulating in the country are unknown. The research reported here was aimed at characterising molecularly HBV in HIV co-infected patients in Botswana to determine the circulating genotypes and to describe the mutations found in the HBV strains.

## Methods

### Study participants

This was a retrospective cross-sectional study. Archived plasma samples from treatment-naïve HIV/HBV co-infected adults were used. A convenient sampling method was employed whereby samples from known hepatitis B positive participants from several previous studies conducted at Botswana Harvard AIDS Institute Partnership were used. Anonymous, unlinked plasma samples from 81 of the participants were available for use in this research.

The study was approved by Human Research Development Committee (HRDC) at Botswana Ministry of Health.

### DNA extraction

DNA was extracted from 200 ul of serum sample using QiAamp DNA Mini Kit according to the manufacturer’s instructions (Qiagen, Hilden, Germany). An elution volume of 50 ul was used. The extracted DNA was directly amplified after extraction or stored at −80 °C until ready for amplification.

### Amplification and sequencing for 415 base pairs

A 415 bp fragment of the surface gene was amplified by semi-nested PCR using Platinum *Taq* DNA Polymerase High Fidelity kit according to manufacturer (Invitrogen ,USA) [39]. The first round had a 5 min denaturing step at 94 °C, and then 30 cycles of denaturing for 45 s at 94 °C, annealing for 30 s at 50 °C, and elongation at 72 °C for 90 s, with extension at 72 °C for 10 min using HBV840 (5′-GTTTAAATGTATACCCAAAGAC-3′;nt840-861) and HBV381 (5′-TGCGGCGTTTTATCATCTTCCT-3′; nt381-402 ) primers [39]. The second round commenced with denaturation at 94 °C for 5 min and then 30 cycles of denaturation at 94 °C for 45 s, annealing at 55 °C for 30 s, elongation at 72 °C for 60 s, and extension at 72 °C for 10 min using HBV381 and HBV801 (5′-CAGCGGCATAAAGGGACTCAAG-3′ nt801-822;) primers [39]. The final product was visualized on 2 % agarose gel stained with ethidium bromide, and the PCR products were purified using QiAquick PCR Purification Kit according to the manufacturer’s instructions (Qiagen, Hilden, Germany). Primers HBV 381 and HBV 801 were used for sequence reactions. Sequencing clean up was done using ZR DNA Sequencing Clean up Kit according to the manufacturer (Zymo, USA). Sequencing of the amplified region was done using Big Dye sequencing chemistry on an ABI 3130xl genetic analyzer (Applied Biosystems, Foster City, CA). Sequences were submitted to National Center for Biotechnology Information (NCBI) Genbank under accession numbers KR139680 to KR139749.

### Amplification and sequencing for 2100 base pairs

For samples with sufficient DNA, a 2100 bp fragment of HBV was amplified by nested PCR using a Taq polymerase kit from Invitrogen (kit name and manufacturer) in a reaction volume of 25 ul. The PCR cycling conditions for both rounds included a 2 min denaturing step at 95 °C, and then 40 cycles of denaturing for 30 s at 95 °C annealing for 30 s at 60 °C, elongation at 72 °C for 4 min, and a final extension of 10 min at 72 °C. The sequences of primers used are given in Table 1. The first round PCR primers were P1 and P2 whilst the second round PCR used the Core F and Werle AS primers to amplify a 2100 bp region of HBV covering amino acid position 1–344 of the polymerase gene. The final product of both rounds was visualized on a 1 % agarose gel stained with ethidium bromide and the samples with amplification were then purified using QIAquick PCR purification Kit from Qiagen. The sequencing was done using Big Dye sequencing chemistry with primers Core F, HBV-3, HBV-N, HBV-P, HBV-Z, HBV-H and P6. A 3130XL ABI sequencer was used to generate the sequences.

**Table 1:** Primers used for PCR and sequencing

Primer Name	Sequence 5′-3′	Position
P1	CCGGAAAGCTTGAGCTCTTCTTTTTCACCTCTGCCTAATCA	1821–1841
P2	CCGGAAAGCTTGAGCTCTTCAAAAAGTTGCATGGTGCTGG-	1823–1806
Werle AS	CGTCAGCAAACACTTGGC	1175–1192
Core F	GTGTGGATTCGCACTCCT	2269–2287
HBV-3	CGTTGCCKDGCAACSGGGTAAAGG	2478–2455
HBV-N	ACTGAGCCAGGAGAAACGGACTGAG GC	1991–1965
HBV-P	TCATCCTCAGGCCATGCAGT	1292–1311
HBV-Z	AGCCCTCAG GCTCAGGGCATA	1179–1199
HBV-H	TATCAAGGAATTCTGCCCGTTTGTCCT	1767–1793
P6	GGCAGGTCCCCTAGAAGAAGAACT	2363–2386

### Analysis of the research results

Sequences were manually edited using Sequencher 5.0, and consensus sequences were formed from the overlapping sequences. The consensus sequences were then uploaded on two online databases: The Stanford HBV database [40] to give the genotypes and resistance mutations, and Geno2Pheno, another online database [41] was used to confirm the genotypes, resistance mutations and to give escape mutations. Phylogenetic analysis was then performed to confirm the genotypes and rule out contamination. Sequences from this research and genotypes A-I references from NCBI Genbank were aligned using ClustalX software version 2.1. The accession numbers for the HBV references were: AB076678, DQ020002, AY2333282, GQ331047, GQ331046, GQ331048, GQ477494, EU594385, FJ904434, AY233276, FM199974, AY233279, FJ692596, FJ692598, FJ692613, HM363612, AM180623, AB194951, HM363613, AB106564, DQ060824, AB048701, JN664938, AJ131956, AJ344117, DQ315779, AB033558, JN664947, AB188245, DQ991753, FJ904439, FJ904405, AB033559, AB048705, AB033556 and AB493838. The Phylogenetic tree was constructed using Maximum Likelihood algorithm implemented in RAxML under the GAMMA model of rate heterogeneity [42, 43]. The statistical support for each node was assessed by bootstrap analysis from 1000 bootstrap replicates performed using the rapid bootstrap algorithm implemented in RaxML version 8 [44]. Pairwise distances in multiple sequence alignments were computed using the Maximum Composite Likelihood model [45] in MEGA 6.06 [46].  Aspartate aminotransferase (AST), alanine aminotransferase (ALT) and AST platelet ratio index (APRI) [47] were compared between genotypes using two sample Wilcoxon rank-sum (Mann–Whitney) test. Participants with escape mutations and those without were also compared using the same test for the said parameters. The threshold of significant fibrosis (APRI ≥1.5) and cirrhosis (APRI ≥2) were used [47]. *P* values <0.05 were considered significant.

## Results

### Characteristics of participants

This research included 81 participants 60 (74 %) of whom were females (Table 2). There were more females because most available samples were from HIV prevention of mother to child transmission cohorts.

**Table 2:** Characteristics of participants

Characteristics		n
Age, median (Q1, Q3) years	32 (26, 40)	81
Gender, female, n (%)	67/81 = (82.7 %)	81
CD4 + T cell count, median (Q1, Q3), cells/mm^3^	241.7 (135.1, 446.5)	78
HIV-1 RNA level, median (Q1, Q3), log10 copies/ml	4.7 (4.3, 5.3)	80
AST, median (Q1, Q3), IU/L	25.6 (19.6, 35.8)	80
ALT, median (Q1,Q3), IU/L	19.8 (11.5, 30.1)	80

### Prevalence of genotypes

**Fig. 1:**
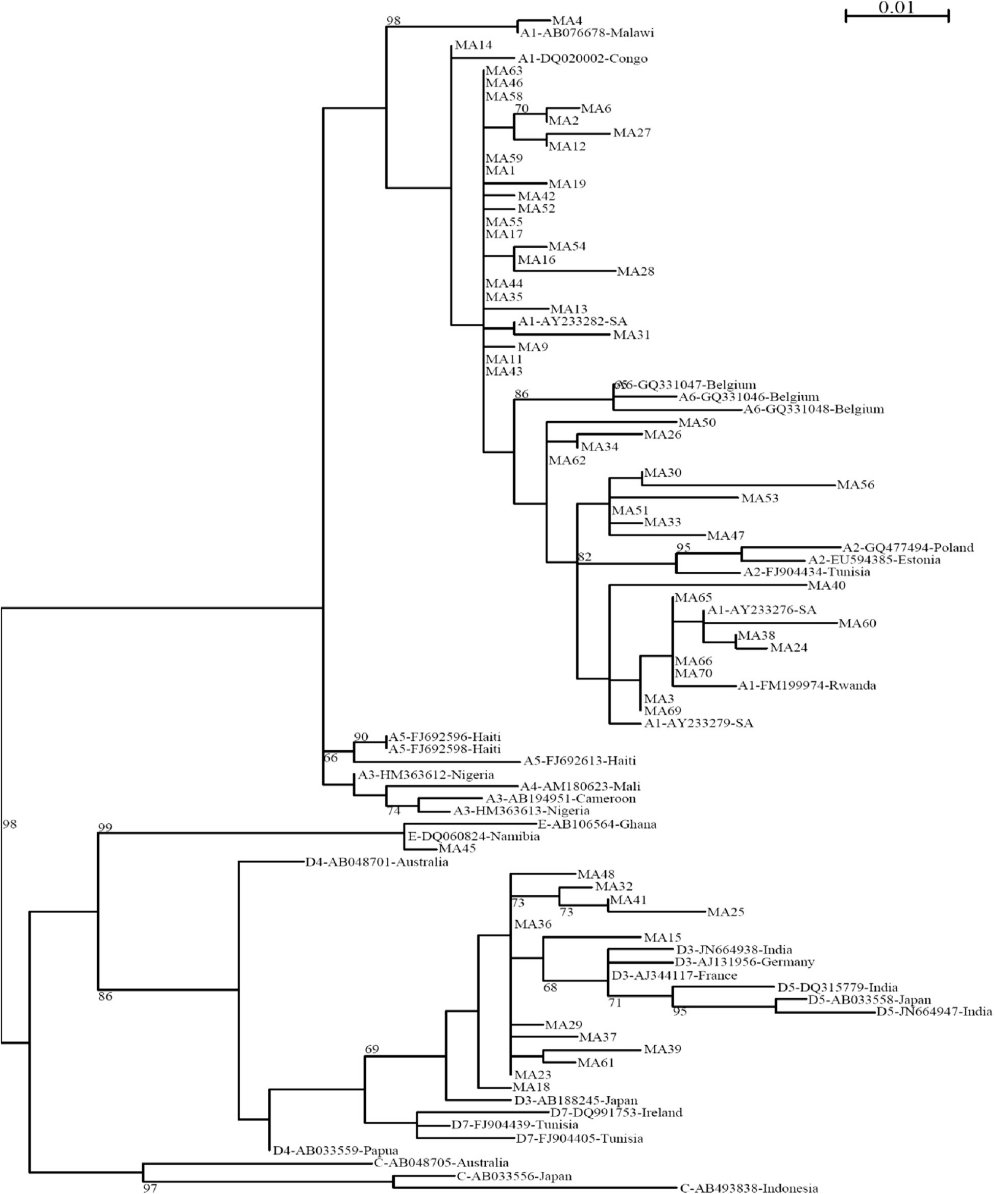
Maximum likelihood phylogenetic tree of Botswana sequences and Genbank HBV references. References names start with subgenotype, accession number and country whereas Botswana genotypes start with MA. The numbers at the nodes represent the percentages of the bootstrap values (1000 replicates)

Of the 81 plasma samples amplified for the 415 bp fragment, 70 (86.4 %) were successfully sequenced. 56 (80 %) were genotype A, whereas 13 (18.6 %) were infected with genotype D, and 1 (1.4 %) participant had genotype E (Fig. 1). For 52 samples, amplification of the 2100 bp fragment was attempted, and 10 (19.2 %) were successfully genotyped, including 6 classified as genotype A, 3 as genotype D, and 1 as genotype E. These genotypes were concordant with those determined using the 415 bp fragment.

The subgenotypes were only determined from the geno2Pheno database, as the 415 bp region was too short to give resolution for the subgenotypes in the phylogenetic tree; however, it is sufficient to determine the genotypes. All A genotypes for the 2100 bp fragment belonged to subgenotype A1, while all the genotype D samples belonged to subgenotype D3. No samples were found to harbor any known HBV drug resistance mutations.

### Prevalence of HBV escape mutations

**Fig. 2:**
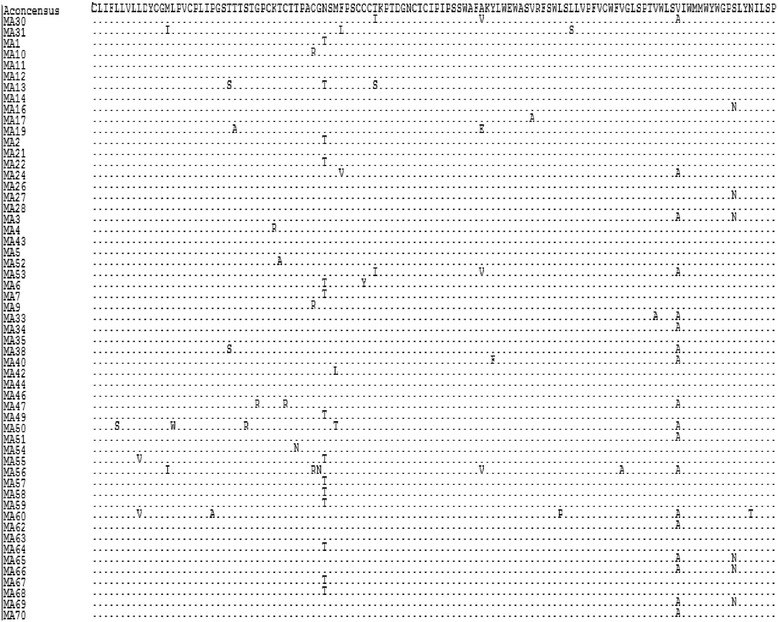
Manual alignments of genotype A and genotype A consensus sequence. Dots indicate identity to the genotype A consensus

**Fig. 3:**
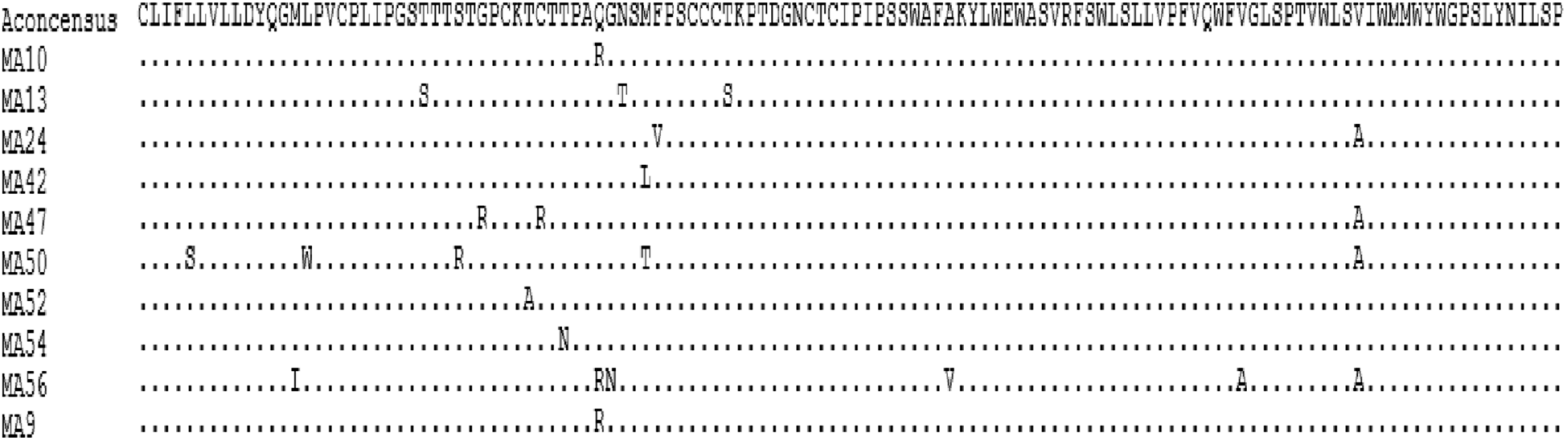
Manual alignments of genotype A escape mutants and genotype A consensus sequence. Dots indicate identity to the genotype A consensus

**Fig. 4:**
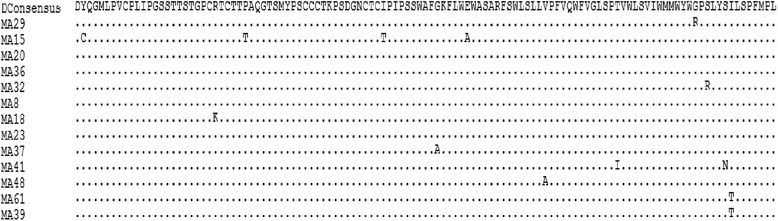
Manual alignments of genotype D and genotype D consensus sequence. Dots indicate identity to the genotype D consensus

**Fig. 5:**
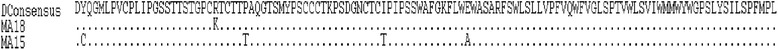
Manual alignments of genotype D escape mutants and genotype D consensus sequence. Dots indicate identity to the genotype D consensus

HBV escape mutations, which were previously reported as associated with either failure of diagnosis, active immunisation or immunoglobulin therapy, were found in 12 (17.1 %) patients. The substitutions detected included Y100C, G119R, R122K, T123A, C124R, T126N, Q129R, G130N, M133T, M133L, F134V and T140S (Table 3). There were eight escape mutations which were previously associated with failure of diagnosis (Y100C, R122K, T123A, C124R, T126A, Q129R, M133T and M133L); three for failure of immunoglobulin therapy (G119R, C124R, and T126N) and four for vaccine escape (T126A, Q129R, M133L and F134V). The escape mutations were also searched for manually in BioEdit (Figs. 2, 3, 4 and 5).

**Table 3:** Escape mutations found in participants and the corresponding genotypes

Mutation	Frequency	Genotype
Y100C	1	D
G119R	1	A
R122K	1	D
T123A	1	A
C124R	1	A
T126N	1	A
Q129R	3	A
G130N	1	A
M133L	1	A
M133T	1	A
F134V	1	A
T140S	1	A

### Factors associated with liver disease severity

Significant liver cirrhosis was present in 3/69 (4.3 %) of which two participants had genotype A and one had genotype D. None of this three participants had escape mutations. Significant liver fibrosis was not found in any of the participants. Genotypes were not significantly associated with AST, ALT and APRI (*p* values: 0.882, 0.773, 0.388 respectively). Having escape mutations was also not significantly associated with AST, ALT, and APRI (*p* values: 0.825, 0.601, 0.160 respectively).

## Discussion

We report here for the first time on the HBV genotypes circulating in HIV/HBV co-infected patients in Botswana and the presence of potential escape mutations. Three HBV genotypes were found at the following frequencies: genotype A (80 %), genotype D (18.6 %), and genotype E (1.4 %). These genotypes are consistent with the literature on the HBV genotypes circulating in the sub-Saharan Africa region [22]. A study in South Africa found the same genotypes (A and D), even though genotype A was still the predominant genotype and the proportions were different (93 % versus 80 %) in Botswana [48]. This difference might be due to the differences in the populations between the two countries.

A study by Scheiblauer *et al.* evaluated performance of multiple HBsAg assays and found that of the 70 kits evaluated, 32 (45.7 %) gave false negative results for some of the S gene escape mutants showing that escape mutations affect the sensitivity of some assays [49]. The mutant M133L was one of those which could not be detected even by one of the polyclonal antibody assays [49] suggesting that recognizing the antigen by the antibodies is more important than the type of diagnostic test [50]. There were other studies which had also demonstrated failure of diagnosis of this mutant [51]. The mutant Q129R which was found in three of the 70 participants has also been associated with diagnostic failure [51], and this might be due to its effect of decreasing surface protein secretion as reported in China [52]. Furthermore the mutant Q129R and M133L have been associated with vaccine escape [53, 54]. A recent study by Forbi *et al.* involving samples from Ghana, Cameroon, Uganda and Ivory Coast also found the mutant M133L in a Cameroon participant and Q129R in a sample from Uganda [55]. However, this study identified fewer escape mutations (7.7 %) [55]. These disparate findings may reflect differences in genotypes [56] as their study had mostly genotype E which was found in only one participant in the present study [55] as genotype E has been found predominantly in West Africa [57]. The research done by Huang *et al.* also associated the mutant C124R with weak reactivity with some of the commercial serology assays [52]. Research done in Poland by Grabarczyk *et al.* detected Q129R, G130N, and M133I mutants [58]. Furthermore, another study in China which utilized 11,221 hepatitis B sequences from NCBI found most of the escape mutations including G130N, M133L, M133T, C124R, T123A, and Q129R with frequencies of mostly >1 % [32]. Mutant F134V has been associated with vaccine escape. The reason some mutations which were previously linked with diagnosis failure were detected in this research might be because of the Murex HBsAg kit which was used has been shown to detect some of the mutants including M133L and Y100C [59]. The most frequently reported vaccine escape mutation, G145R was not found in this population similar to what has been reported in Oman [60] but in contrast to other studies [55].

The current study is the first to report molecular characterization of HBV in Botswana, a high HBV endemic area. Hepatitis B genotype D was found in 13 of the 70 participants (18.6 %) which might mean some of the HBV patients in Botswana might not respond to interferon therapy. Some escape mutations which were previously linked with escaping diagnosis, passive and active hepatitis B immunization were detected. Some diagnostic kits have difficulty detecting some genotypes like Genotype D and some S gene mutants; therefore, it is important to know the molecular characteristics of the circulating HBV in the population especially in areas were blood transfusion screening is done only by the use of serological assays as in Botswana [49]. No baseline resistance mutations were found hence all oral antiviral drugs might be effective in this population.  This study found no significant difference in APRI, a non invasive test which has been recommended by WHO for staging of liver disease in resource limited settings [47], between genotypes and escape mutations. These results concur with a multicentre study which was done in France [61] and is in contrast  to what has been reported by Lacombe *et al.* [62]. Significant liver cirrhosis was found in 4.3 % and none of the participants had significant liver fibrosis according to the recent WHO guidelines [47].

## Conclusion

The HBV genotypes found circulating in Botswana were genotypes A (80 %), D (18.6 %) and E (1.4 %). These data are important in guiding future treatment strategies since 18.6 % of HBV-infected people might not respond to certain antiviral treatment like interferon therapy.

Escape mutations which were previously associated with failure of diagnosis, vaccine and hepatitis B immunoglobulin therapy escape were also observed. The information on escape mutations is important when developing diagnostic tests, vaccines and hepatitis B immunoglobulin therapy. They lower efficiency of the HBV vaccine, increase rates of liver disease and pose a risk of transmission to others through blood transfusion where only serological assays are used in blood screening.

The molecular characteristics of hepatitis B virus have been reported for the first time in Botswana. The hepatitis B virus molecular characterization work is important to inform policy makers when developing future diagnostic tests and strategies, vaccines and immunoglobulin therapy and choosing antiviral therapy which will be most effective for the population. No baseline resistance mutations were found; hence all oral antiviral drugs might be effective in this population. The genotypes and escape mutations were not significantly associated with stage of liver disease in this study.

The limitations of this research are that the sample size is relatively small, and it only included the HIV-positive participants which were mostly women; thus, these data may not be generalizable to other populations. There might be other HBV variants in the population which were not picked up in this research. Only the S gene escape mutations were determined but there are other clinically important mutations in other genes of the virus.

There is a need in future to generate data in a larger sample size which can be extrapolated to the general population. Future work is also needed to determine the clinical significance of genotypes and escape mutations in Botswana and also to amplify other genes of the Hepatitis B virus.
